# Community pharmacist-led point-of-care eGFR screening: early detection of chronic kidney disease in high-risk patients

**DOI:** 10.1038/s41598-024-56765-0

**Published:** 2024-03-27

**Authors:** Ammar Abdulrahman Jairoun, Sabaa Saleh Al-Hemyari, Moyad Shahwan, Samer H. Zyoud, Faris El-Dahiyat

**Affiliations:** 1 Health and Safety Department, Dubai Municipality, Dubai, United Arab Emirates; 2 Discipline of Clinical Pharmacy, School of Pharmaceutical Sciences, Universiti Sains Malaysia (USM), 11800 George Town, Pulau Pinang Malaysia; 3 Pharmacy Department, Emirates Health Services, Dubai, United Arab Emirates; 4 Centre of Medical and Bio-allied Health Sciences Research, Ajman University, 346, Ajman, United Arab Emirates; 5 Department of Clinical Sciences, College of Pharmacy and Health Sciences, Ajman University, 346, Ajman, United Arab Emirates; 6 Department of Mathematics and Sciences, Ajman University, P.O. Box 346, Ajman, United Arab Emirates; 7 Clinical Pharmacy Program, College of Pharmacy, Al Ain University, 64141 Al Ain, United Arab Emirates; 8 AAU Health and Biomedical Research Center, Al Ain University, 112612 Abu Dhabi, United Arab Emirates

**Keywords:** Chronic kidney disease, Community pharmacy, Directed point-of-care, Screening program, eGFR, Kidney, Kidney diseases, Medical research, Nephrology

## Abstract

Adherence to scheduled physician screenings for renal function monitoring in patients with chronic kidney disease (CKD) or those at high risk remains suboptimal despite the endorsement of regular screenings by several clinical practice guidelines. Our study aims to assess the effectiveness of a point-of-care CKD screening program led by these pharmacists using the PICCOLO device while recognizing the unique position of community pharmacists in primary care. We conducted an 11-month prospective point-of-care interventional research study in the United Arab Emirates to evaluate the performance of a community pharmacist-led CKD screening program for high-risk patients. Six diverse community pharmacies were selected based on staff availability, patient volume, and their offered range of services. Eligible individuals with risk factors for CKD were identified during medication evaluations. The PICCOLO Comprehensive Metabolic Panel facilitated on-site blood analysis, delivering estimated Glomerular Filtration Rate (eGFR) results within 10 to 15 min. Data also included eGFR categories, demographic information, and insights into lifestyle and health habits collected through a questionnaire. Pharmacists conducted comprehensive medication reviews and offered referrals and lifestyle guidance as part of the program. The study encompassed a total of 400 patients, with an average age of 69 ± 13.4 years within the study cohort. Notably, 38.8% (155 individuals) of the 400 patients were found to have undiagnosed CKD stages 3–5. Univariate logistic regression analysis revealed a significant association between a higher incidence of CKD stages 3–5 and factors such as older age, a history of hypertension, vascular disease, and diabetes mellitus. In the multivariate regression model, age and a history of diabetes mellitus emerged as significant predictors of an elevated risk of CKD. This study sheds light on the viability and impact of CKD screening programs conducted by community pharmacists, particularly in detecting CKD stages 3–5. The findings have implications for healthcare policies, as they can influence the enhancement of early detection and management of CKD. Moreover, these insights may catalyze focused screening initiatives and strengthen collaboration between community pharmacies and healthcare systems to benefit patients at high risk of CKD.

## Introduction

According to the classifications established by the Kidney Disease Improving Global Outcomes (KDIGO) and the Kidney Disease Outcomes Quality Initiative (KDOQI), there is a global increase in chronic kidney disease (CKD), impacting roughly 3% to 18% of the world's population^1^. CKD is characterized by irreversible renal damage or loss in sustained kidney function lasting longer than 3 months, often progressing slowly over months to years^2^. It is typically identified by a permanent decrease in renal function, defined as an estimated glomerular filtration rate (eGFR) < 60 mL/min/1.73 m^2^, or the presence of other markers of kidney damage, such as albuminuria ≥ 3 mg/mmol, abnormalities in urine sediment, or renal imaging anomalies, all persisting for more than 3 months^1^. Furthermore, CKD significantly contributes to elevated rates of morbidity and mortality in the general population, serving as an independent risk factor for cardiovascular disease and hypertension^1^.

In the last two decades, there has been a notable increase in the global occurrence of CKD, impacting 13.4% of the global population, primarily concentrated in stages 3–5 of the disease^3^. Recent data indicate that 2.8% of females and 4.6% of males in the United Arab Emirates (UAE) are in these advanced stages of CKD^4^. These statistics might only represent conservative approximations, especially considering the elevated prevalence of well-documented CKD risk factors within the United Arab Emirates (UAE)^5^.

In the UAE, hypertension (HTN) has witnessed a significant increase over the last 20 years, emerging as a major contributor to CKD^6–8^. Additionally, type 2 diabetes mellitus (DM), another significant risk factor^9,10^, affects 29% of UAE citizens^11^. Furthermore, the population of the United Arab Emirates exhibits higher than average rates of smoking, dyslipidemia, and obesity—all well-known cardiovascular risk factors—than people in wealthier countries^12,13^. It is well-recognized that these modifiable cardiovascular risk factors influence the rate of CKD progression^14–18^, particularly when several risk factors coexist^19–21^.

Due to the rarity or non-specificity of early symptoms, 90% of cases go undetected^22^. This allows the disease to progress into later stages silently, often only detected when renal failure becomes acute or when patients reach stage 5 CKD (eGFR < 15 mL/min)^22,23^. Advanced CKD stages not only necessitate hemodialysis or transplantation but can also bring about symptoms such as weight loss, vomiting, anorexia, pruritus, or muscle cramps. Complications of CKD and the subsequent end-stage renal disease (ESRD) encompass anemia, bone abnormalities, a heightened risk of cardiovascular disease, and an increased risk of all-cause mortality^23^.

Early intervention is the key to reducing the morbidity and mortality linked to chronic kidney disease (CKD)^24^. Yet, the silent nature of CKD in its initial stages raises concerns about the feasibility of timely detection as recommended by clinical guidelines. In primary care, many patients either receive insufficient treatment or remain undiagnosed^25^. Therefore, a critical imperative is to conduct focused and easily accessible screening, particularly for high-risk individuals, including those with diabetes, hypertension, a family history of kidney disease, or cardiovascular disease^26^. In this context, community pharmacists assume a unique and pivotal role in delivering targeted Chronic Kidney Disease (CKD) screening, effectively serving as the vanguard of primary care. A notable initiative in this regard is the application of the CKD Clinical Pathway, previously outlined^26^. The efficacy of this strategy has been assessed in Alberta, Canada^27^. Under their authority, pharmacists conducted, ordered, and interpreted laboratory tests, as demonstrated in the study by Al Hamarneh et al.^27,28^. Impressively, 720 high-risk patients were successfully screened for CKD following the CKD Clinical Pathway, revealing that 40% of the individuals examined had CKD, with 40% of this subgroup previously undiagnosed^27^.

While the incidence of CKD in the UAE and other Arab nations remains unknown^29^, establishing local data is paramount to comprehending the epidemiological aspects of CKD in the high-risk population. The primary objective of this study is to ascertain the prevalence of chronic kidney disease (CKD) among high-risk patients by evaluating the effectiveness of a point-of-care screening program led by community pharmacists utilizing the PICCOLO device. Furthermore, the study aims to identify the CKD risk factors most predictive of previously undetected renal function impairment within the community pharmacy setting, serving as its secondary goal.

## Methods and materials

### Study design and setting

This prospective point-of-care interventional study spanned from January 1st to November 30th, 2023, with the primary aim of investigating the prevalence of chronic kidney disease (CKD) in high-risk patients and evaluating the effectiveness of a point-of-care screening program administered by community pharmacists. The study was conducted at six carefully selected community pharmacies in the United Arab Emirates, encompassing both chain and independent establishments, and participation was voluntary and random.

The choice of these pharmacies was deliberate, based on specific criteria. They were chosen for their substantial patient volume and the comprehensive range of services, including medication reviews, management of chronic diseases, prescription medication dispensing, recommendations for over-the-counter (OTC) medications, as well as nutrition and dietary counseling. Additionally, these pharmacies had the requisite personnel capable of conducting the study with precision and competence.

### Study population (inclusion and exclusion criteria)

During the process of medication evaluation, individuals, both UAE nationals and non-UAE nationals, were considered eligible for participation if they exhibited at least one of the following recognized risk factors for chronic kidney disease (CKD): diabetes, hypertension, a history of cardiovascular illness, a family history of renal disease, or an age above 55 years.

To be eligible for participation in the study, individuals had to meet the following criteria: Aged 18 or over and meet at least one of the following conditions under the KDIGO International Guidelines, rendering them at risk for CKD:

- Diabetes mellitus (either type 1 or type 2).
- Hypertension.
- A documented family history of kidney disease.

### Sample size calculation

Given a reported CKD prevalence of 4.6% among males and 2.8% among females in the UAE^3^ and considering the recent study's findings revealing an 11.4% incidence of CKD stages 3–5^30^, we initially anticipated that the percentage of individuals with CKD stages 3–5 in our study would approximate 12%.. Our chosen significance level (alpha) was 5% to produce 95% confidence intervals. Moreover, we aimed for a precision (D) of 5% within these 95% confidence intervals to ensure a broad 95% range of ± 10%.

Based on these underlying assumptions, we determined that a minimum sample size of n = 312 participants would be necessary, factoring in an estimated nonresponse rate of approximately 50%. Consequently, our final sample size was established at 400 participants.

### Data collection

Patients visiting the pharmacy for prescription drop-off or pick-up were identified as potential participants. Those who met the eligibility criteria were then approached and invited to join the study, receiving comprehensive information about its purpose and procedures. Subsequently, they were requested to provide written informed consent indicating their willingness to participate.

The pharmacy's staff pharmacists oversaw the consent process and facilitated utilizing the on-site PICCOLO Comprehensive Metabolic Panel and blood chemistry analyzer for data collection. Once the necessary data had been gathered, additional consent was sought from patients to allocate a unique participant ID, ensuring both confidentiality and anonymity in the use of their data for research purposes. Patients were allowed to share their demographic and result information anonymously voluntarily.

### Intervention and measurement tool

A disposable lancet was employed to extract a venous blood sample through a self-administered fingerstick, following the prior cleaning of the finger with an alcohol swab.

To prevent significant hemolysis, the initial blood drop was carefully wiped away. Subsequently, a minimum sample volume of 100 μL was acquired through capillary action and gentle finger pressure at the puncture site, allowing for the collection of successive drops into a designated collection tube. Following this, the pharmacist pipetted the collected material into a metabolic panel disc to prepare it for analysis using the PICCOLO instrument. Once the blood was placed within the panel disc, it underwent heparinization and was spun into cuvette wells that contained dry sample blank reagent beads. These beads were equipped with the necessary reagents, buffers, surfactants, and excipients for absorption chemistry analysis^32^.

The PICCOLO blood chemistry analyzer utilized the Chronic Kidney Disease Epidemiology Collaboration (CKD-EPI) research equation and the creatinine levels assessed by the panel to estimate the Glomerular Filtration Rate (eGFR) for each patient, incorporating the provided age, gender, and race data^31,32^. Typically, results were available within a span of 10 to 15 min.

### PICCOLO instrument

The PICCOLO device, when deployed at the point of care, serves as a portable diagnostic analyzer offering a comprehensive array of CLIA-waived blood chemistry tests. CLIA-waived blood tests are those exempt from the Clinical Laboratory Improvement Amendments (CLIA) requirements in the United States. Irrespective of the testing location, the CLIA federal regulatory framework establishes quality standards for all laboratory testing, ensuring precision, reliability, and the swift delivery of patient test results. Tests deemed straightforward with a low risk of inaccurate results and those considered user-friendly fall under the waived category.

The PICCOLO instrument represents an innovative, fully automated tool employed at the point of care across diverse healthcare settings, including pediatric offices, oncology clinics, urgent care centers, and physician's offices. In blood testing, CLIA-waived tests are typically uncomplicated point-of-care procedures that can be conducted outside traditional laboratory settings.

Meeting a wide range of clinical chemistry needs, the PICCOLO Xpress delivers real-time blood chemistry diagnostic information within minutes. Despite its compact size, this PICCOLO instrument excels in accuracy, reliability, and repeatability.

These tests are designed for individuals without laboratory backgrounds, such as healthcare professionals working in clinics, pharmacies, or other point-of-care settings. They often require minimal technical expertise. Depending on their waiver status, certain more stringent regulatory requirements applicable to more complex laboratory processes are waived for these tests.

### Study variables

An estimated Glomerular Filtration Rate (eGFR) of 90 or higher indicated normal renal function. An eGFR falling within the range of 60 to 89 (CKD Stage 2) signified a mild impairment in renal function, while an eGFR in the 30 to 59 range (CKD Stage 3) indicated a significant reduction in renal function.

Each participant completed a paper-based questionnaire during this phase, providing additional insights into the specific risk factor for which they were selected to participate. This questionnaire collected data on demographics (age, gender), lifestyle factors, and health characteristics (such as tobacco use, body mass index [BMI], history of diabetes, hypertension, kidney disease, vascular disease, and family history of renal disease). Additionally, the pharmacist conducted a comprehensive medication review with each patient and made tailored recommendations, which might include dietary adjustments or referrals to their primary care physician when necessary. These recommendations were grounded in the patient's medication history and risk factors, relying on the pharmacist's professional judgment.

### Ethical approval

The study received approval from the Institutional Ethical Review Committee of Ajman University (Approval Number: P-H-S-2022-2-9). All methods were carried out under relevant guidelines and regulations. Before collecting data, all participants were duly informed about the study's objectives and explicitly consented to completing the questionnaire. Written informed consent was obtained from all respondents. To safeguard participant anonymity, no information was gathered that could potentially disclose their identities, and rigorous measures were implemented to uphold the confidentiality of participant data.

### Statistical analyses

For data analysis, we utilized SPSS Version 26. Continuous variables with a normal distribution were assessed using means and standard deviations (SD), while categorical variables were analyzed through frequencies and percentages. We employed one-way ANOVAs, nonparametric alternatives, and unpaired Student t-tests to identify disparities among quantitative variables. In order to determine the variables that exerted an influence on CKD, we applied univariate and multivariate logistic regression models. Statistical significance was defined when p < 0.05.

## Results

### Demographics and comorbidities of the study cohort

A total of 400 patients were enrolled in the study. The mean age of the study cohort was 69 ± 13.4 years. The mean eGFR was 76.3 ± 27 mL/min/1.73 m^2^. Of the total, 43% (n = 172) were male and 10% (n = 40) were smokers. The comorbidities among the study cohort were as follows: vascular disease (n = 180, 45%), Hypertension (n = 346, 86.5%) and Diabetes Mellitus (n = 129, 32.3%) (Table 1).

**Table 1:** Demographics and comorbidities of the study cohort.

Demographics and comorbidities	Mean (SD)/n (%)
Age (years), mean (SD)	69 (13.4)
BMI, mean (SD)	30.34 (7.1)
eGFR (mL/min/1.73 m^2^), mean (SD)	76.3 (27)
Male gender n (%)	172 (43)
Smoking n (%)	40 (10)
Vascular disease n (%)	180 (45)
Hypertension n (%)	346 (86.5)
Diabetes mellitus n (%)	129 (32.3)

*eGFR* estimated glomerular filtration rate.

### Prevalence of unrecognized CKD by community pharmacist-directed point-of-care screening program

Table 2 displays the prevalence of various CKD stages categorized by patients' demographics and comorbidities. A statistically significant difference in eGFR levels was observed based on age (P < 0.001), vascular disease (P = 0.037), hypertension (P = 0.001), and diabetes mellitus (P < 0.001).

**Table 2:** Prevalence of CKD stages according to patient's demographics and comorbidities.

Demographics and comorbidities	eGFR					*P* value
	≥ 90	60–89	45–59	30–44	< 30	
Age (years), mean (SD)	60.65 (11.6)	69.35 (11.5)	76.2 (11)	79.1 (11.3)	80.6 (12.1)	< 0.001
BMI, mean (SD)	31.34 (7)	30.23 (7)	29.5 (7.5)	29.43 (7.8)	28.35 (5.7)	0.220
Male gender n (%)	60 (34.9)	42 (24.4)	50 (29.1)	15 (8.7)	5 (2.9)	0.942
Smoking n (%)	13 (32.5)	13 (32.5)	13 (32.5)	1 (2.5)	0	0.304
Vascular disease n (%)	79 (43.9)	46 (25.6)	39 (21.7)	12 (6.7)	4 (2.2)	0.037
Hypertension n (%)	117 (33.8)	82 (23.7)	105 (30.3)	32 (9.2)	10 (2.9)	0.002
Diabetes mellitus n (%)	26 (20.2)	31 (24)	55 (42.6)	11 (8.5)	6 (4.7)	< 0.001

In our study cohort of 400 patients, 155 patients had unrecognized/undiagnosed CKD (38.8%). The results of univariate logistic regression revealed an increased prevalence of CKD stages 3–5 associated with older age (OR 1.099, 95% CI 1.075–1.123, p < 0.001), a history of hypertension (OR 1.66, 95% CI 1.015–2.73, p = 0.043), a history of vascular disease (OR 1.528, 95% CI 1.349–1.798, p = 0.002), a history of hypertension (OR 4.25, 95% CI 1.95–9.27, p < 0.001), and a history of diabetes mellitus (OR 2.86, 95% CI 1.85–4.41, p = 0.001) (Table 3).

**Table 3:** Univariate and Multivariate regression analysis for the factors for developing CKD stages 3–5.

Factors	eGFR < 60							
	Univariable				Multivariable			
	OR	95% CI		p-value	OR	95% CI		p-value
Age	1.099	1.075	1.123	< 0.001	1.090	1.064	1.116	< 0.001
BMI	0.969	0.939	0.999	0.053	1.0	0.963	1.037	0.983
Gender (ref. female)								
Male	1.155	0.770	1.732	0.487	1.003	0.595	1.692	0.991
Smoking (ref. no)								
Yes	0.836	0.422	1.656	0.608	1.002	0.411	2.445	0.996
Vascular disease (ref. no)								
Yes	1.528	1.349	1.798	0.002	1.675	0.910	1.109	0.121
Hypertension (ref. no)								
Yes	4.25	1.95	9.27	< 0.001	1.837	0.738	4.572	0.191
Diabetes mellitus (ref. no)								
Yes	2.861	1.856	4.411	< 0.001	2.095	1.260	3.485	0.004

In the multivariate regression model, after a backward stepwise selection, the significant predictors that increased the risk of CKD stages 3–5 included older age (OR 1.090, 95% CI 1.064–1.116, p < 0.001) and a history of diabetes mellitus (OR 2.095, 95% CI 1.260–3.485, p = 0.004) (Table 3).

*eGFR* estimated glomerular filtration rate.

*eGFR* estimated glomerular filtration rate.

## Discussion

Compliance with scheduled physician screenings for renal function remains alarmingly inadequate, with rates fluctuating between a mere 28% and a modest 75%, despite the advocacy of numerous clinical practice guidelines for regularly monitoring chronic kidney disease (CKD) in CKD patients and those at elevated risk^33^. Community pharmacies, with their accessibility and convenience, hold promise as potential game-changers, motivating a more significant number of patients to undergo testing^34^. Anecdotal feedback from pharmacists has highlighted the value of CKD screening for patients, the successful integration of such screening into their workflow, and its role in setting their pharmacies apart in a competitive landscape.

Notably, this study breaks new ground by examining the effectiveness of a point-of-care screening program led by community pharmacists for early detection of abnormal renal function in high-risk patients, employing an on-site PICCOLO Comprehensive Metabolic Panel and blood chemistry analyzer. Our research underscores the feasibility and viability of implementing point-of-care testing for CKD screening within a community pharmacy context. In the course of our investigation, it came to light that 155 patients (38.8%) exhibited CKD stages 3–5, a condition that had not previously been recognized or diagnosed. This contrasts with earlier studies in diverse populations and nations, where undiagnosed CKD was comparatively less prevalent. For instance, Donovan et al. reported an 11% incidence of CKD, with 90% of cases going undetected^35^.

Similarly, Arora et al. found that 12.5% of adult Canadians had CKD, based on data from the Canadian Health Measures Survey^36^. Our findings align with prior research conducted both in community and laboratory settings^22,37^. Additionally, the SeeKD study 2016 revealed that 18.8% of 6329 patients with at least one CKD risk factor had undiagnosed CKD^38^.

Using a multivariate regression model and employing a backward stepwise selection process, we pinpointed that a history of diabetes mellitus and advancing age consistently emerge as significant predictors for a heightened risk of developing CKD stages 3–5. These robust associations underscore the importance of considering age and diabetes mellitus history when assessing CKD risk, in line with findings from previous studies^36^. As a result, our findings strongly support the adoption of tailored interventions and screening tactics, especially among older demographics and those with a background of diabetes mellitus. These measures are pivotal in improving the timely identification and control of CKD. These identified risk factors underscore the need for proactive healthcare practices and public health campaigns to raise awareness and promote preventative measures within populations at risk for chronic kidney disease. This aligns with the emphasis placed on such strategies by Al Hamarneh and colleagues, who utilized laboratory testing to screen high-risk individuals for CKD^27^.

The implications of this research extend into optimizing medication regimens and developing strategies to mitigate the progression of CKD by addressing risk factors, including cardiovascular events. These findings have profound implications for patient care^39–41^. Despite the robust evidence supporting pharmacists' involvement in laboratory testing^28^, it is worth noting that this practice has not been widely adopted across most jurisdictions^35^.

The current study successfully demonstrated the feasibility of implementing a pharmacist-guided point-of-care screening program for chronic kidney disease (CKD) within a community pharmacy setting. Additionally, our study has uncovered a significant proportion of previously undiagnosed CKD cases. Given the frequent misdiagnosis and inadequate treatment of CKD, the role of pharmacists in its detection and management is paramount. This has far-reaching implications for enhancing patient access to care, prognostic outcomes, cardiovascular risk management, and drug dosage optimization. Furthermore, it underscores the indispensable role of pharmacists within the broader spectrum of pharmacy practice.

In the context of community pharmacy, our study sheds light on the prevalence and risk factors associated with CKD stages 3–5. The substantial percentage of our study participants with previously undetected CKD (38.8%) underscores the critical importance of adopting proactive screening initiatives, especially among older adults and those with a history of diabetes mellitus.

Our findings emphasize the significance of diabetes mellitus and age as independent predictors of CKD stages 3–5, even considering other comorbid and demographic factors. This highlights the imperative need for targeted interventions and educational programs aimed at these high-risk populations, ultimately enhancing the early identification and management of CKD.

The utilization of point-of-care testing within a community pharmacy setting underscores the potential extension of screening services beyond traditional healthcare settings. This approach emphasizes the valuable contribution of community pharmacies to public health initiatives, aligning with the overarching goal of promoting preventive healthcare.

However, it is important to acknowledge the limitations of this research. The possibility of selection bias during pharmacist recruitment and the requirement for patients to provide informed consent may have excluded individuals with known CKD or those unwilling to participate. Additionally, potential recall bias may exist since demographic data relied on self-reporting and dispensing history. The absence of follow-up at the end of the 3 months poses a challenge in confirming CKD, as CKD is a condition that evolves over time, and diagnosis requires multiple eGFR readings. Furthermore, the assessment of the impact of pharmacist interventions on patients with low eGFR was constrained by the lack of follow-up. For a more precise evaluation, the KDIGO 2012 guideline recommends categorizing CKD based on both GFR and albumin levels^33^. Unfortunately, the pharmacy's inability to collect and analyze urine samples precluded the categorization of patients according to the KDIGO criteria.

## Conclusion

Our research offers significant insights into the occurrence and determinants of advanced CKD stages (3–5) within the context of community pharmacies. This valuable information has the potential to guide healthcare policies, promote focused screening initiatives, and strengthen the collaborative partnership between community pharmacies and healthcare systems. Ultimately, these efforts aim to enhance the early detection and effective management of CKD.

## Data Availability

The original contributions presented in the study are included in the further inquiries that can be directed to the corresponding authors.
